# Comparative analysis of the burden of young-onset and late-onset dementia in China from 1990 to 2021: A study based on GBD 2021 data

**DOI:** 10.1016/j.tjpad.2025.100307

**Published:** 2025-07-24

**Authors:** Ke-qiang Lu, Ke-jia Lu, Zheng-jun Ji

**Affiliations:** a Institute of Liver Diseases, Shuguang Clinical Medical College, Shanghai University of Traditional Chinese Medicine, Shanghai 201203, PR China; b Ko Chi Ming Centre for Parkinson's Disease Research (CPDR), School of Chinese Medicine, Hong Kong Baptist University, Hong Kong SAR, PR China; c Institute for Research and Continuing Education, Hong Kong Baptist University, Shenzhen, PR China; d Department of Neurology, Xuzhou Cancer Hospital, Xuzhou, PR China

**Keywords:** Young-onset dementia, Late-onset dementia, Temporal trend, Disease burden

## Abstract

**Background:**

Most epidemiological studies on dementia in China have focused on the elderly population, with a lack of systematic comparisons between the burden of young-onset dementia (YOD) and late-onset dementia (LOD).

**Methods:**

Based on data from the Global Burden of Disease (GBD) study, this research systematically evaluated changes in the burden of YOD and LOD in China over different time periods. The analysis employed average annual percentage change (AAPC), Bayesian age-period-cohort (BAPC) modeling, decomposition analysis, risk factor attribution analysis, health inequality analysis, and frontier analysis.

**Results:**

AAPC analysis showed that the growth rate of YOD has significantly outpaced that of LOD since 2012. Forecasting results indicated that the age-standardized rates for both YOD and LOD are expected to continue rising in the future. Decomposition analysis revealed that between 1990 and 2021, the main drivers of the increasing YOD burden shifted from population growth to epidemiological changes and population aging, whereas population growth remained the dominant driver for LOD. Risk factor analysis indicated that the impact of high BMI on both YOD and LOD has become increasingly pronounced. Health inequality and frontier analyses suggested that, although disparities in YOD and LOD burden across different SDI regions were not significant, there remains substantial room for improvement in managing both conditions in China.

**Conclusion:**

In recent years, YOD has exhibited a more rapid increase compared to LOD, with its driving forces gradually shifting from population-related factors to epidemiological transitions. This highlights the need to strengthen identification and intervention strategies targeting younger and middle-aged populations. Tobacco use, high fasting plasma glucose, and high BMI are key modifiable risk factors shared by both YOD and LOD, with particular attention needed on the sustained impact of high BMI. Although international disparities in health inequality are not pronounced, China still holds considerable potential for improvement in the prevention and control of both YOD and LOD. Future interventions should be more forward-looking, systematic, and tailored to specific population groups.

## 1 Introduction

Dementia refers to a significant decline in cognitive abilities that impairs an individual’s capacity to independently perform daily activities. It can be regarded as a syndrome caused by various primary neurological disorders, neuropsychiatric conditions, and other medical factors, often resulting from the combined effects of multiple diseases [1].

Based on the age of onset, dementia can be classified into young-onset dementia (YOD), which occurs before the age of 65, and late-onset dementia (LOD), which occurs at or after the age of 65^2^. Regardless of the subtype, the diagnosis of dementia imposes a long-term psychological, social, and economic burden on both patients and their families, underscoring an urgent need for comprehensive intervention and support.

With the ongoing deepening of population aging in China, the burden of age-related diseases has become increasingly pronounced. Since China entered an aging society in 2000, the proportion of individuals aged 65 and older has steadily increased, reaching 14.2 % by the end of 20,21^3^. This demographic shift has directly driven the rapid increase in the burden of age-related neurodegenerative diseases, such as LOD, thereby posing substantial challenges to the public health system, healthcare resource allocation, and the social security infrastructure.

Compared to LOD, which is strongly associated with aging, patients with YOD are diagnosed at a younger age, typically during middle adulthood, and thus do not belong to the traditional elderly population category. Diagnosis of YOD largely depends on passive monitoring systems and is often delayed due to limited awareness among clinicians[4,5]. Moreover, individuals with YOD are usually at critical stages of their careers and family responsibilities. The disease not only compromises individual health but also reduces productivity and disrupts social functioning, thereby imposing broader socio-economic consequences. Therefore, despite lacking the typical "age-related" characteristics, YOD warrants significant attention from public health policymakers.

China bears the highest number of dementia cases globally; nevertheless, existing epidemiological research has predominantly concentrated on the general older population, with study participants typically limited to individuals aged 55 or 60 and above[[6], [7], [8]]. This research paradigm—treating dementia as a homogeneous condition without differentiating between subtypes—not only contributes to the underrepresentation of YOD in policymaking and clinical practice, but also obscures critical differences in disease burden between YOD and LOD. As a result, it hinders the formulation and implementation of targeted, stratified intervention strategies. At present, there is a notable absence of nationwide studies in China that specifically examine the differential burden of disease between YOD and LOD, underscoring the urgent need for large-scale data analyses to address this research gap.

Within the framework of the Global Burden of Disease 2021 (GBD 2021), disease burden is defined as a comprehensive measure of the negative impact of a disease on individuals and society, typically quantified using epidemiological indicators such as incidence, prevalence, mortality, and disability-adjusted life years (DALYs). In this study, the “differential burden of disease between YOD and LOD” refers to differences in the levels and temporal trends of these indicators between YOD and LOD. Based on publicly accessible GBD 2021 data, this study performs a secondary analysis to systematically compare the evolving patterns of disease burden related to YOD and LOD in China from 1990 to 2021. The analysis period was divided into three phases: 1990–2000, 2001–2010, and 2011–2021. By accounting for shifts in population aging, the study examines the dynamic trajectories of these two dementia types and aims to provide theoretical evidence to support the development of more targeted, age-specific prevention strategies.

## 2 Materials and methods

### 2.1 Overview

In the GBD database, dementia is categorized under Alzheimer’s disease and other dementias (ADODs). Given that available data primarily pertain to populations aged 40 and above, this study defines YOD as ADODs-related data for individuals aged 40–64, and LOD as data for those aged 65–95 and older. The GBD analysis adopts reference case definitions based on the *Diagnostic and Statistical Manual of Mental Disorders* (DSM series, including DSM-III, DSM-IV, and DSM-5) and the *International Classification of Diseases* (ICD series, including ICD-8, ICD-9, and ICD-10). DSM criteria are primarily used in surveys and cohort studies, while ICD codes are more commonly applied in mortality registration and health claims data. For the identification of ADODs in the GBD framework, specific ICD codes are employed: ICD-9 codes include 290, 291.2, 291.8, 294, and 331; ICD-10 codes include F00, F01, F02, F03, G30, and G319[9].

### 2.2 Data acquisition

According to GBD 2021, data on ADODs in China from 1990 to 2021 were obtained from the Global Health Data Exchange (https://ghdx.healthdata.org/gbd-results-tool). Prevalence, deaths, and DALYs were extracted from the “Cause of Death and Injury” module, and related risk factors (tobacco use, high fasting plasma glucose, and high body mass index) were obtained from the “Risk Factors” module. The location selected was China (excluding Taiwan Province of China).

### 2.3 Data analysis

To effectively compare the disease burden of YOD and LOD, this study calculated the age-standardized rates of ADODs-related indicators for the 40–64 and 65–95+ age groups using the $\text{ASR}=\frac{\sum_{i=1}^{n}\left(w_{i}\cdot r_{i}\right)}{\sum_{i=1}^{n}w_{i}}\times \;100,000$ formula [10].In the formula, *ri* represents the age-specific rate for the *i* age group.*wi* represents the population of the same age group based on the GBD 2021 standard population, and n denotes the total number of age groups.

This study measured the temporal trends of YOD and LOD using the Average Annual Percent Change (AAPC) model and projected future trends through the Bayesian Age-Period-Cohort (BAPC) model.

Decomposition analysis is used to evaluate the contribution of different factors to changes in disease burden. Each factor can act as either a driving factor (positive contribution), which increases the disease burden, or a suppressing factor (negative contribution), where certain changes (such as population size or disease prevalence) buffer the overall disease burden and help mitigate the burden increase caused by other factors (such as population aging).

Specifically, both the AAPC and decomposition analyses conducted separate evaluations for the sub-period of 2017 to 2021 to capture short-term changes potentially influenced by recent public health initiatives in China related to dementia prevention and the promotion of cognitive health among the elderly.

Health inequality was assessed using the concentration index, a measure ranging from –1 to +1 that quantifies socioeconomic-related inequality in disease burden. Negative values indicate concentration among lower-income groups, positive values among higher-income groups, and zero indicates perfect equality.

Frontier analysis was performed to evaluate China’s relative performance in managing YOD and LOD burden compared to countries with similar sociodemographic development levels. This approach estimates the minimum achievable burden given the country’s development status and quantifies the gap between observed and optimal performance, highlighting potential improvement space.

All data processing in this study was conducted using R version 4.3.3.

## 3 Result

### 3.1 Trends in AAPC of YOD and LOD in China from 1990 to 2021

The disease burden of YOD and LOD in China from 1990 to 2021 was calculated based on established formulas. The AAPC across five time periods (1990–2021, 1990–1999, 2000–2010, 2012–2021, and 2017–2021) was estimated using the Joinpoint regression model. Results indicated that, over the entire period from 1990 to 2021, LOD showed higher AAPC values across all indicators compared to YOD.

Specifically, from 1990 to 2021, the AAPC for YOD was 0.27 (95 % CI: 0.24–0.31) for DALYs, 0.02 (95 % CI: –0.03–0.05) for number of deaths, 0.90 (95 % CI: 0.81–0.95) for incidence, and 0.64 (95 % CI: 0.59–0.68) for prevalence. In contrast, the AAPC for LOD during the same period was 0.88 (95 % CI: 0.81–0.93) for DALYs, 1.08 (95 % CI: 1.04–1.12) for number of deaths, 1.98 (95 % CI: 1.82–2.23) for incidence, and 1.38 (95 % CI: 1.26–1.47) for prevalence.

In contrast, during the period from 2017 to 2021, the AAPCs for YOD surpassed those for LOD in all indicators except for deaths. Specifically, the AAPC for YOD was 1.38 (95 % CI: 1.30–1.47) for DALYs, 0.72 (95 % CI: 0.31–1.04) for number of deaths, 2.35 (95 % CI: 1.65–2.84) for incidence, and 1.82 (95 % CI: 1.71–1.96) for prevalence. In the same period, the AAPC for LOD was 0.92 (95 % CI: 0.30–1.33) for DALYs, 0.76 (95 % CI: 0.58–0.89) for number of deaths, 1.15 (95 % CI: 0.34–1.63) for incidence, and 1.42 (95 % CI: 0.54–1.94) for prevalence.

After dividing the period from 1990 to 2021 into four distinct stages, the AAPC results demonstrated an overall upward trend over time in all indicators for both YOD and LOD. Detailed results are presented in Table 1.

**Table 1:** AAPC results for different periods.

Period	Measure	YOD		LOD	
		AAPC	Pvalue	AAPC	Pvalue
1990–2021	DALYs	0.27 (0.24 to 0.31)	<0.01	0.88 (0.81 to 0.93)	<0.01
1990–1999	DALYs	-1.13 (-1.23 to -1.01)	<0.01	0.94 (0.89 to 0.99)	<0.01
2000–2009	DALYs	0.26 (0.16 to 0.34)	<0.01	0.94 (0.9 to 0.99)	<0.01
2012–2021	DALYs	1.38 (1.3 to 1.47)	<0.01	0.76 (0.48 to 0.88)	<0.01
2017–2021	DALYs	1.38 (1.3 to 1.47)	<0.01	0.92 (0.3 to 1.33)	<0.01
1990–2021	Deaths	0.02 (-0.03 to 0.05)	<0.01	1.08 (1.04 to 1.12)	<0.01
1990–1999	Deaths	-1.52 (-1.63 to -1.42)	<0.01	1 (0.89 to 1.08)	<0.01
2000–2009	Deaths	0.23 (0.14 to 0.32)	<0.01	1.43 (1.29 to 1.59)	<0.01
2012–2021	Deaths	0.97 (0.83 to 1.07)	<0.01	0.76 (0.59 to 0.89)	<0.01
2017–2021	Deaths	0.72 (0.31 to 1.04)	<0.01	0.76 (0.58 to 0.89)	<0.01
1990–2021	Incidence	0.9 (0.81 to 0.95)	<0.01	1.98 (1.82 to 2.23)	<0.01
1990–1999	Incidence	0.18 (0.07 to 0.28)	<0.01	-0.72 (-0.92 to-0.51)	<0.01
2000–2009	Incidence	0.37 (0.17 to 0.51)	<0.01	0.92 (0.81 to 1.13)	<0.01
2012–2021	Incidence	1.9 (1.59 to 2.07)	<0.01	0.94 (0.58 to 1.15)	<0.01
2017–2021	Incidence	2.35 (1.65 to 2.84)	<0.01	1.15 (0.34 to 1.63)	<0.01
1990–2021	Prevalence	0.64 (0.59 to 0.68)	<0.01	1.38 (1.26 to 1.47)	<0.01
1990–1999	Prevalence	-0.51 (-0.66 to -0.35)	<0.01	2.08 (1.91 to 2.37)	<0.01
2000–2009	Prevalence	0.22 (0.07 to 0.35)	<0.01	1 (0.87 to 1.2)	<0.01
2012–2021	Prevalence	1.82 (1.71 to 1.95)	<0.01	1.12 (0.71 to 1.33)	<0.01
2017–2021	Prevalence	1.82 (1.71 to 1.96)	<0.01	1.42 (0.54 to 1.94)	<0.01

### 3.2 Prediction of the disease burden related to YOD

**Fig. 1:**
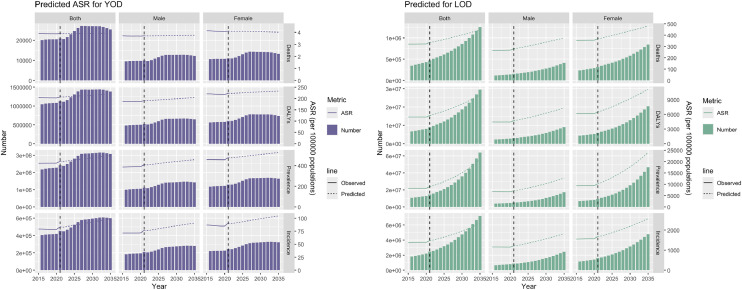
Predicted results for YOD and LOD, including deaths, DALYs, incidence, and prevalence.

This study employed the BAPC forecasting model to analyze the future trends of YOD and LOD in China. The results showed that, in terms of age-standardized rates (ASR), all indicators—except for the ASR of YOD-related deaths—are projected to continue rising through 2035 for both YOD and LOD, with this trend remaining consistent across sexes. In terms of absolute numbers, indicators for YOD are expected to stabilize after a period of growth, whereas those for LOD are projected to continue increasing in line with the ASR trends, again consistently across both sexes (see Fig. 1).

### 3.3 Decomposition analysis of the disease burden related to YOD

**Fig. 2:**
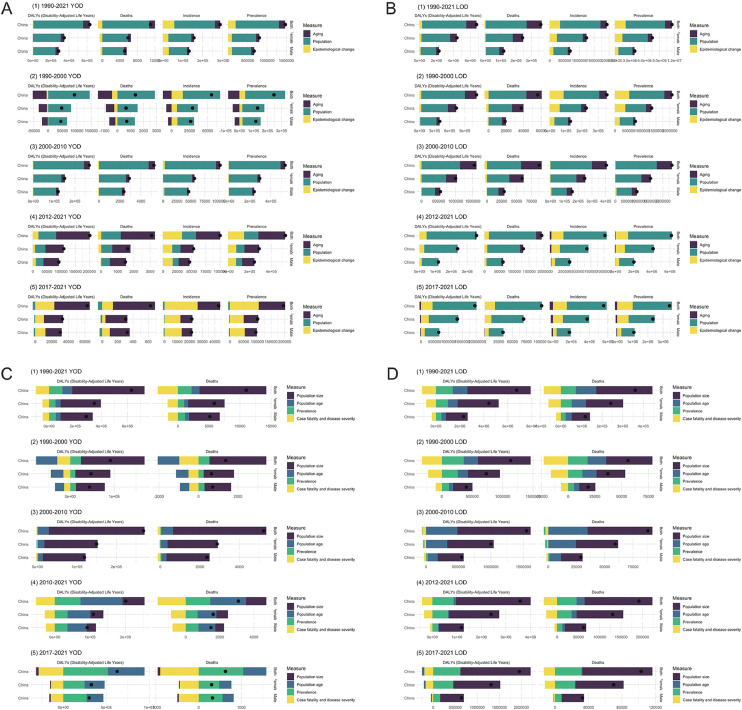
Decomposition analysis results: A. Three-way division for YOD, B. Three-way division for LOD, C. Four-way division for YOD, D. Four-way division for LOD.

To gain a more comprehensive understanding of the primary drivers behind the changes in disease burden of YOD and LOD from 1990 to 2021, this study employed a decomposition analysis approach. It assessed the contributions of key factors—including population growth, population aging, and epidemiological changes—to the evolution of YOD- and LOD-related indicators. The analysis results indicate that, from 1990 to 2021, population growth was the primary driver of the increasing disease burden for both YOD and LOD. Looking at different time periods, for YOD, population growth was the main driving factor from 1990 to 2010. By contrast, between 2012 and 2021, population aging gradually became the predominant factor. Specifically, during the 2017–2021 period, the increase in YOD burden was primarily driven by both population aging and epidemiological changes (see Fig. 2A). In contrast, for LOD, population growth remained the primary driving factor throughout the entire 1990–2021 period (see Fig. 2B). This trend was largely consistent across different sexes.

In addition to the aforementioned approach, this study also utilized a complementary decomposition framework to analyze the contributions of population size, population age, prevalence, case fatality and disease severity to the changes in DALYs and deaths attributable to YOD and LOD. Given that prevalence was regarded as one of the primary drivers, the analysis was limited to DALYs and mortality figures.

The results show that from 1990 to 2021, population size was the primary driver of the increased disease burden for both YOD and LOD. Improvements in case fatality and disease severity have somewhat slowed the upward trend in disease burden. For YOD, population size remained the main contributor from 1990 to 2010. From 2012 to 2021, population aging and changes in prevalence emerged as the dominant drivers. Between 2017 and 2021, the increase in YOD burden was almost entirely driven by population aging and rising prevalence, with population size even acting as a suppressing factor (see Fig. 2C).

For LOD, population size remained the predominant driver of disease burden throughout the study period. Between 2012 and 2021, prevalence emerged as a secondary contributing factor. Notably, during the period from 2017 to 2021, population age suppressed the growth of LOD-related DALYs (see Fig. 2D). This trend was consistent across different sexes.

### 3.4 Risk factor analysis

**Fig. 3:**
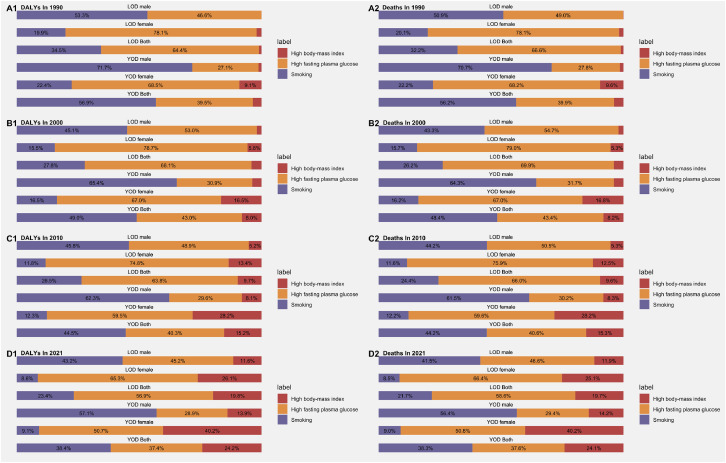
Proportion of risk factors for deaths and DALYs related to LOD and YOD at multiple time points.

According to the GBD 2021 database, the DALYs and deaths related to YOD and LOD were primarily attributed to three major risk factors: tobacco, high fasting plasma glucose, and high body mass index. This study focuses on the years 1990, 2000, 2010, and 2021 to analyze the changes in the proportion of these risk factors for YOD and LOD in China. The results indicate that over time, the impact of tobacco on YOD and LOD has gradually decreased, while the proportion of impact from high body mass index has increased. Notably, tobacco use has a greater impact on males compared to females for both YOD and LOD, whereas the impact of high fasting plasma glucose and high body mass index is higher in females than in males (Fig. 3).

### 3.5 Health inequality analysis

To further understand China's international position and equity in terms of YOD and LOD disease burden, this study analyzed the distribution differences of YOD and LOD burden across countries with different SDI levels based on the 1990–2021 GBD data. The concentration index (CIX) was calculated to assess the inequity of the burden based on national social development levels.

The concentration index results showed that in 1990, the concentration index for LOD-related DALYs was 0.13 (95 % CI: 0.11, 0.14), and in 2021, it was 0.13 (95 % CI: 0.12, 0.15). For LOD-related deaths, the concentration index in 1990 was 0.16 (95 % CI: 0.14, 0.18), and in 2021, it remained 0.16 (95 % CI: 0.14, 0.18).

**Fig. 4:**
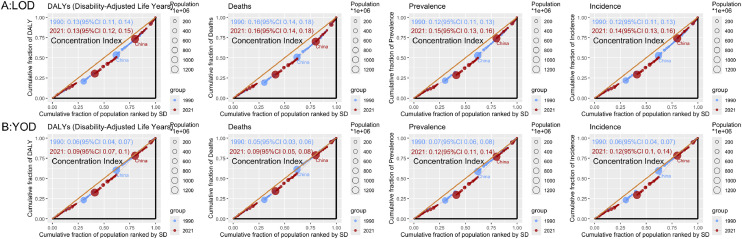
Concentration index results. Blue represents data from 1990, and red represents data from 2021. A: LOD, B: YOD.

In 1990, the concentration index for ADODs-related prevalence was 0.12 (95 % CI: 0.11, 0.13), and in 2021, it increased to 0.15 (95 % CI: 0.13, 0.16). For ADODs-related incidence, the concentration index in 1990 was 0.12 (95 % CI: 0.11, 0.13), and in 2021, it was 0.14 (95 % CI: 0.13, 0.16) (Fig. 4A). The concentration indices for YOD and ADODs are as follows: In 1990, the concentration index for YOD-related DALYs was 0.06 (95 % CI: 0.04, 0.07), and in 2021, it increased to 0.09 (95 % CI: 0.07, 0.10). For YOD-related deaths, the concentration index in 1990 was 0.05 (95 % CI: 0.03, 0.06), and in 2021, it rose to 0.09 (95 % CI: 0.05, 0.08). For ADODs-related prevalence, the concentration index was 0.07 (95 % CI: 0.06, 0.08) in 1990 and 0.12 (95 % CI: 0.11, 0.14) in 2021. For ADODs-related incidence, the concentration index in 1990 was 0.06 (95 % CI: 0.04, 0.07), and in 2021, it increased to 0.12 (95 % CI: 0.10, 0.14) (Fig. 4B).

### 3.6 Frontier analysis

**Fig. 5:**
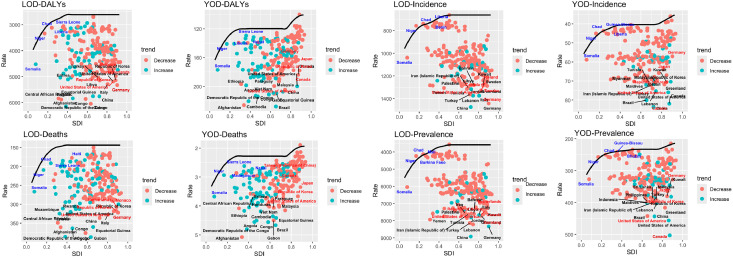
Frontier analysis results for all countries in 2021. Countries marked in black are the top fifteen countries with the largest effective gap globally. Countries marked in blue are the five countries with the smallest effective gap in low and middle SDI regions, while countries marked in red are the five countries with the largest effective gap in middle and high SDI regions.

To assess China's potential for improvement in the control of YOD and LOD, this study conducted a frontier analysis based on data from the GBD database for various countries (Fig. 5). The analysis measured the effective gap (efficiency gap) between the actual disease burden and the theoretical optimal values to reflect the potential for improvement in disease control.

The results show that, except for the death count related to YOD, there are significant efficiency gaps in various indicators for YOD and LOD in China in 2021. Specifically, for YOD, China's effective gap for DALYs was 87.6, ranking 11th globally; for deaths, the effective gap was 1.63, ranking 23rd; for prevalence, it was 232.97, ranking 3rd; and for incidence, it was 43.31, ranking 2nd. In the case of LOD, the effective gap for DALYs was 3152.69, ranking 5th globally; for deaths, it was 192.69, ranking 8th; for prevalence, it was 5326.15, ranking 1st; and for incidence, it was 811.62, also ranking 1st

## 4 Discussion

The World Health Organization (WHO) data show that dementia has risen to the 5th leading cause of death in China in 2021[11]. Over 100 epidemiological studies in China have estimated the incidence and prevalence of dementia[6]. However, these studies typically focus on restricted age groups and often do not distinguish between YOD and LOD, resulting in the long-term marginalization of the YOD population in disease monitoring and public health strategies.

Given the significant differences in patient characteristics and care needs between YOD and LOD, this study conducted a systematic secondary analysis of dementia-related disease burden data in China using the GBD database from 1990 to 2021. To our knowledge, this is the first study to comprehensively compare the disease burden characteristics of YOD and LOD. Utilizing methodologies including AAPC analysis, decomposition analysis, risk factor assessment, health inequality analysis, and frontier analysis, this study reveals the multidimensional differences in disease burden between YOD and LOD. The findings provide critical evidence for characterizing the epidemiological profiles of YOD and LOD and offer valuable insights to optimize health resource allocation and enhance the precision of policy interventions.

This study calculated the AAPC of YOD- and LOD-related indicators in China from 1990 to 2021 across multiple time intervals (Table 1). Overall, both YOD and LOD showed an increasing trend in disease burden throughout the study period, with YOD exhibiting a comparatively slower growth rate. However, a more detailed decade-specific analysis of AAPC revealed an accelerating increase in the burden of YOD, particularly during 2012–2021, when the disease burden of YOD surpassed that of LOD.

The decadal variations in the AAPC for YOD may be attributed to the increasing volume of YOD-related research publications over time. The accelerated growth in YOD burden is likely linked to this expanding body of literature, particularly following the pivotal 2010 Lancet Neurology publication by Rossor et al., which formally defined YOD as dementia with onset before the age of 65[2]. This definition established a theoretical framework that has since underpinned subsequent epidemiological, social care, and mechanistic studies. Further influential contributions, including Deborah A. Levine’s work in JAMA Internal Medicine and publications by David S. Knopman and Stevie Hendriks in JAMA Neurology, have collectively heightened multidisciplinary awareness of YOD. These landmark studies have significantly advanced understanding, thereby fostering increased research activity and shaping policy discourse around young-onset dementia[4,12,13].

Compared to YOD, LOD has consistently garnered greater attention in China. As previously noted, over 100 epidemiological studies have focused primarily on LOD. Consequently, despite a surge in public awareness, the disease burden of LOD has not exhibited the same rapid growth as that of YOD. Notably, the AAPC values for LOD incidence and prevalence were higher during the 2017–2021 period than in 2012–2021, suggesting an accelerated rate of change in these two indicators in the past five years. To better anticipate the future trajectory of YOD and LOD disease burdens in China, this study employed the BAPC model for predictive analysis.

The projection results suggest that, based on trends from 1990 to 2021, the rates of key indicators for both YOD and LOD are expected to continue rising steadily in the future. However, it is important to emphasize that this forecast reflects the average trajectory over the past three decades and may underestimate the recent acceleration in YOD burden observed over the last ten years.

Notably, the AAPC values for the periods 2012–2021 and 2017–2021 indicate that the incidence and prevalence of YOD have been increasing at a faster rate than those of LOD. If this upward trend persists in the absence of robust intervention strategies, the momentum of YOD burden is likely to intensify further, posing escalating challenges to public health systems.

The overall results of the decomposition analysis reveal that, for both YOD and LOD, the primary driver of the increasing disease burden is population growth (Fig. 2).

For instance, in the case of LOD, although China entered an aging society in the early 21st century and the aging trend continued to intensify through 2021, the decomposition analysis indicates that the contribution of population aging (i.e., the increasing proportion of older adults) to the rising disease burden is less significant than the "base effect" associated with overall population expansion. This suggests that in developing countries with large and rapidly growing populations, addressing the health challenges posed by population growth remains a crucial priority in shaping future public health strategies. Nevertheless, since 2021, China has entered a phase of population decline, while the trend of population aging is projected to continue intensifying[3,14]. This implies that, moving forward, the primary driving force behind LOD in China may gradually shift toward population aging. Establishing the “Healthy Ageing” system advocated by the WHO will become a key strategy for addressing the continued increase in LOD burden[15]. In this context, Giulia Grande and colleagues outlined three core strategies for preventing LOD during the aging process: (1) implementing physical health interventions to preserve brain function; (2) adopting compensatory approaches to delay or mitigate brain aging; and (3) promoting lifelong health behaviors such as maintaining an active lifestyle, quitting smoking, and following a balanced diet[16].These strategies offer valuable practical guidance for developing countries seeking to prevent and manage LOD in the face of population aging.

In contrast, the decomposition analysis results for YOD indicate that its disease burden became predominantly driven by changes in age structure earlier than that of LOD. Another notable factor is the increasing role of epidemiological change, which appears to be closely linked to the gradual rise in public awareness of YOD in recent years. This growing awareness has led to heightened clinical attention and continuous advances in diagnostic technologies, a trend expected to persist and expand in the coming years[[17], [18], [19], [20], [21]]. While this may contribute to an increase in the documented burden of YOD, it also creates an important opportunity to advance early detection and implement evidence-based preventive strategies—developments that are encouraging from a public health perspective.

Despite these positive shifts, research on the rising risk factors specific to YOD remains relatively limited. It remains unclear whether the risk factors identified for LOD are equally applicable to YOD, underscoring the need for further investigation. For example, Dr. Stevie Hendriks conducted a study using data from the UK Biobank to examine potential modifiable risk factors for YOD [22]. The study identified 15 risk factors associated with YOD, some of which differed from those commonly linked to LOD. Notably, the findings revealed that moderate or heavy alcohol consumption was less strongly associated with YOD than complete abstinence, whereas individuals diagnosed with alcohol use disorder showed a significantly elevated risk of developing YOD.

Building upon the exploration of modifiable risk factors, this study further reveals that while YOD and LOD share several common risk factors, notable sex-based differences exist in their distribution and impact. These findings underscore the importance of incorporating sex-specific considerations into the design of intervention strategies.

For instance, the impact of tobacco use is predominantly observed among males. Notably, Chinese men consume about 40 % of the world’s cigarettes, with smoking rates among men ranking among the highest globally. Over 300 million people in China smoke, while approximately 740 million non-smokers are exposed to secondhand smoke[23,24]. As early as 1998, a study by A. Ott et al., published in *The Lancet*, highlighted that smoking is associated with nearly a twofold increase in dementia risk[25]. In light of the extensive health hazards posed by smoking, the WHO has intensified global tobacco cessation efforts through the Framework Convention on Tobacco Control (FCTC) and the "MPOWER" tobacco control measures[26].

China has ratified the Framework Convention on Tobacco Control (FCTC) and implemented local tobacco control regulations in several provincial capitals and municipalities. Nevertheless, significant regional disparities exist in the enforcement of these measures. For example, Shanghai demonstrates relatively strong enforcement; however, the male smoking prevalence there was still 34.8 % in 2018, the lowest rate nationwide[27]. This situation underscores the urgent need to establish unified national tobacco control legislation and to ensure its effective implementation across all regions.

Existing studies indicate that diabetes, including prediabetes, and related changes in biochemical markers, are associated with an increased risk of cognitive impairment and dementia[28]. In this study, the impact of hyperglycemia on dementia-related DALYs and mortality is predominantly observed in the female population. Further research suggests that although blood glucose control in female patients with type 2 diabetes mellitus (T2DM) tends to be better than in males, women experience more pronounced hippocampal volume loss, implying greater susceptibility to diabetes-related brain damage[29]. Additional studies have shown that female T2DM patients are more likely to develop comorbidities such as cerebral infarction, brain atrophy, and other neurological complications[30,31]. These findings provide further support for the sex differences identified in this study.

With the acceleration of the global nutritional transition, obesity has become an increasingly prominent public health concern. In recent years, numerous studies have investigated the association between BMI and dementia. Some research indicates that increased adiposity during middle age may serve as an independent risk factor for dementia or Alzheimer’s disease, whereas in older age, a decline in BMI may act as an early warning sign of dementia[[32], [33], [34]]. In a 40-year longitudinal study, Jinlei Li and colleagues examined the relationship between BMI changes from middle age to late life and the risk of dementia. Their findings showed that individuals with an overall decrease in BMI were more likely to develop dementia. Further analysis revealed that those who experienced a BMI increase during early middle age followed by a decrease in late middle age had a particularly elevated risk of dementia[35]. These findings underscore the significant impact of BMI fluctuations at different life stages on cognitive health. Moreover, some studies suggest that improving physical fitness and maintaining a healthy weight during childhood can enhance cognitive function in middle age. Conversely, other research argues that there is no clear causal relationship between childhood obesity and Alzheimer’s disease[36,37].

According to data from the World Health Organization, the population with high BMI in China continues to increase[38]. Given the extensive impact of elevated BMI on a range of chronic diseases, including dementia, the Chinese government has issued the *China Obesity Prevention and Control Blue Book* to establish comprehensive strategies for obesity prevention and managementl[39]. The findings of this study indicate that the influence of high BMI on dementia-related DALYs and mortality in China has been steadily rising year by year. A local study from Sweden reported an association between higher BMI and lower mortality among dementia patients; however, this study primarily focused on the elderly population and did not include individuals with YOD[40]. Similarly, a regional study in China found that low BMI was linked to increased dementia mortality in those over 60 years old, while emphasizing that individuals with elevated BMI should aim to maintain their weight within a normal range[41]. The effect of high BMI on dementia outcomes may be mediated through its interplay with chronic conditions such as diabetes, hypertension, and cardiovascular diseases. Therefore, maintaining a healthy body weight is crucial not only for preventing the onset of dementia but also for potentially delaying disease progression and reducing mortality risk.

The combined use of health inequity analysis and frontier analysis elucidates the relative status and improvement potential of YOD and LOD within China’s global disease burden context. The health inequity analysis indicates that the disease burden of YOD and LOD is somewhat concentrated in regions with high SDI, although the differences are not pronounced (Fig. 4). Conversely, frontier analysis demonstrates that China still holds substantial potential for health gains related to YOD and LOD, implying that even at the current level of resources and development, there is room to further mitigate the disease burden (Fig. 5). These results offer quantitative support for optimizing intervention strategies and resource allocation, thereby guiding policy development toward greater efficiency.

This study has several limitations. First, it relies on estimates from the GBD database. While the GBD offers consistent and internationally comparable data, it lacks estimates for individuals under 40 years old in the context of YOD, which may lead to an underestimation of the true burden. Second, this study does not differentiate between dementia subtypes, which may mask heterogeneity in risk profiles. Lastly, the associations between risk factors and dementia observed in this study are ecological in nature and cannot establish causality; future research using individual-level data is necessary to confirm these findings.

## 5 Conclusion

This study systematically compared the disease burden characteristics of YOD and LOD in China using GBD data, revealing both shared and distinct influencing factors and temporal trends. AAPC and decomposition analyses indicate that individuals with YOD require enhanced comprehensive screening and diagnostic support, whereas the increased burden among LOD populations over recent decades has been primarily driven by population growth. Looking forward, greater attention must be given to the resource demands and management challenges posed by an evolving aging population structure. Tobacco use, fasting hyperglycemia, and high BMI were identified as key risk factors for both YOD and LOD, with tobacco use exerting a more pronounced effect in males, while metabolic factors were more prominent among females. These findings underscore the need to broaden the scope of healthy aging to include younger age groups in dementia prevention and control, thereby enabling more comprehensive, population-based intervention and management strategies.

## CRediT authorship contribution statement

**Ke-qiang Lu:** Writing – original draft, Visualization, Software, Methodology, Data curation. **Ke-jia Lu:** Writing – original draft, Visualization, Methodology. **Zheng-jun Ji:** Writing – review & editing, Methodology, Formal analysis.

## Declaration of competing interest

The authors declare that there are no conflicts of interest regarding the publication of this paper.

## Appendix Supplementary materials

Image, application 1
