# Supplementary material for: Comparative analysis of the burden of young-onset and late-onset dementia in China from 1990 to 2021: A study based on GBD 2021 data
Source: J Prev Alzheimers Dis. 2025 Jul 24;12(9):100307. doi: 10.1016/j.tjpad.2025.100307 (PMC12501349; doi:10.1016/j.tjpad.2025.100307)
Supplement: Supplementary file 1 [file mmc1.docx]

***Trend Analysis Using Joinpoint Regression***

We applied a Joinpoint regression model, a widely used epidemiological method for analyzing temporal trends in disease incidence and mortality, to estimate the Annual Percentage Change (APC) and Average Annual Percentage Change (AAPC) along with their 95% confidence intervals (CI) ^[1]^. A statistically significant upward trend is indicated when both the AAPC estimate and the lower bound of its 95% CI are greater than zero. A statistically significant downward trend is confirmed when both the AAPC estimate and the upper bound of its 95% CI are less than zero. If neither condition is met, the age-standardized rate (ASR) is considered stable over the study period ^[2]^.

***Development Forecast***

The Bayesian Average Annual Percentage Change (BAPC) forecasting model is an extension of the APC model that utilizes Bayesian statistical methods. This model incorporates historical data patterns, uncertainty factors, and prior knowledge, allowing it to effectively handle noise and ambiguity in the data, resulting in more reliable predictions.^[3]^

***Decomposition Analysis of ADOD Burden Drivers***

To quantify the contributions of different factors to ADOD burden changes from 1990 to 2021, we conducted decomposition analysis using two complementary approaches: 1) Demographic Decomposition: This approach partitions changes in ADOD burden into three key components: population aging, population growth, and epidemiological transition; 2) Epidemiological Decomposition: This method further disaggregates disease burden changes into four factors: population size, population age structure, disease prevalence, and case fatality rate/severity. These methodologies enable a granular assessment of the underlying drivers of ADOD burden evolution and facilitate cross-national comparisons ^[4-5]^.

***Frontier Analysis for Benchmarking Disease Burden Reduction***

We applied frontier analysis, a quantitative method for evaluating the minimum achievable disease burden at a given level of socioeconomic development. Using SDI and age-standardized mortality/DALYs rates, we delineated a disease burden frontier, which represents the theoretically lowest burden attainable at different development levels ^[6]^. By computing the effective difference between each country's observed burden and this frontier, we identified gaps in disease control efficiency and potential opportunities for burden reduction.

***Health Inequality Analysis Using Socioeconomic Indicators***

To assess disparities in ADOD burden across population subgroups, we conducted health inequality analysis using statistical indices such as Slope Index of Inequality (SII) and Concentration Index (CIX). These metrics quantify the extent to which socioeconomic status, geographic location, gender, and age influence ADOD burden over time. We analyzed trends from 1990 to 2019, providing insights into the evolving impact of socioeconomic disparities on health outcomes ^[7]^.

1. Joinpoint Trend Analysis Software Version 4.9.1.0-April 2022; Surveillance Research Program, Division of Cancer Control & Population Sciences, National Cancer Institute. <https://surveillance.cancer.gov/joinpoint/>.
2. Tuo Y, Li Y, Li Y, et al. Global, regional, and national burden of thalassemia, 1990-2021: a systematic analysis for the global burden of disease study 2021. *EClinicalMedicine.* 2024;72:102619.
3. Hu W, Fang L, Zhang H, Ni R, Pan G. Global disease burden of COPD from 1990 to 2019 and prediction of future disease burden trend in China. Public Health. 2022; 208:89-97. doi: 10.1016/j.puhe.2022.04.015
4. Xu T, Wang B, Liu H, et al. Prevalence and causes of vision loss in China from 1990 to 2019: findings from the Global Burden of Disease Study 2019. *Lancet Public Health.* 2020;5(12):e682-e691.
5. Mortality GBD, Causes of Death C. Global, regional, and national life expectancy, all-cause mortality, and cause-specific mortality for 249 causes of death, 1980-2015: a systematic analysis for the Global Burden of Disease Study 2015. *Lancet (London, England).* 2016;388(10053):1459-1544.
6. Xie Y, Bowe B, Mokdad AH, et al. Analysis of the Global Burden of Disease study highlights the global, regional, and national trends of chronic kidney disease epidemiology from 1990 to 2016. *Kidney Int.* 2018;94(3):567-581.
7. Organization WH. Handbook of statistical methods for public health (ISBN: 9789241548632). WHO Team, Data, Analytics & Delivery for Impact (DDI). <https://www.who.int/publications/i/item/9789241548632>.
